# First- and Second-Trimester Uterine Artery Doppler and Hypertensive Disorders in Twin Pregnancies

**DOI:** 10.3390/jcm14155563

**Published:** 2025-08-07

**Authors:** Stephanie Springer, Teresa Anzböck, Katharina Worda, Eva Karner, Christof Worda

**Affiliations:** 1 Department of Obstetrics and Gynecology, Medical University of Vienna, 1090 Vienna, Austria; stephanie.springer@meduniwien.ac.at (S.S.); katharina.worda@meduniwien.ac.at (K.W.); eva.karner@meduniwien.ac.at (E.K.); 2 Department of Obstetrics and Gynecology, University Hospital Bonn, 53127 Bonn, Germany; teresa.anzboeck@ukbonn.de

**Keywords:** preeclampsia, twin pregnancies, uterine artery, Doppler, adverse pregnancy outcome, pulsatility index, UTPI

## Abstract

**Objective:** The objective of this study is the investigation of uterine artery Doppler studies in twin pregnancies. **Methods:** This retrospective cohort study included 554 twin pregnancies. All women underwent measurement using the mean uterine artery pulsatility index (UTPI) in gestational weeks 11^+0^–13^+6^ and 19^+0^–22^+6^ for risk assessment regarding the occurrence of preeclampsia and adverse obstetric outcomes. **Results:** Out of the 554 included women, a total of 51 women (9.2%) developed preeclampsia: 12 women (2.2%) developed early preeclampsia and 39 patients (7.0%) developed late preeclampsia. Adverse pregnancy outcomes occurred in 147 women (26.5%). The optimum cut-off for the mean UTPI to predict preeclampsia was calculated for gestational weeks 11^+0^–13^+6^ (UTPI > 1.682) and 19^+0^–22^+6^ (UTPI > 1.187). Between gestational weeks 11^+0^ and 13^+6^, the risk of developing preeclampsia was approximately 1.5 times higher when the mean UTPI was above the established cut-off. The risk of early preeclampsia increased 2.5-fold, and that of adverse pregnancy outcomes increased 1.5-fold. At 19^+0^ to 22^+6^ weeks, the preeclampsia risk doubled when the mean UTPI exceeded the cut-off. The risk increased 4-fold for early preeclampsia and 1.5-fold for adverse pregnancy outcomes. Regression analyses revealed that a mean UTPI above the set cut-off at both time points was significantly associated with preeclampsia, early preeclampsia, and adverse pregnancy outcomes. **Conclusions:** The best prediction for early preeclampsia can be achieved using a two-tailed screening approach that combines mean UTPI measurements taken at gestational weeks 11^+0^–13^+6^ and 19^+0^–22^+6^.

## 1. Introduction

A substantial increase in twin pregnancies has been observed over the past few decades [1,2,3,4,5,6]. It is well known that multiple pregnancies are associated with higher obstetric complication rates, which compromises both the fetuses and mother [7,8].

It is well documented that pregnancy-related hypertensive disorders constitute a substantial cause of fetal and maternal morbidity and mortality globally [9,10]. Several studies have highlighted an increased risk of pregnancy-related hypertensive disorders in multifetal gestations [11,12,13,14,15]. The probability of pregnancy-related hypertensive disorders positively correlates with the number of fetuses carried out, and these disorders affect approximately 6.5% of all singleton pregnancies, 12.7% of twin pregnancies, and 20% of all triplet pregnancies [16]. Preeclampsia (PE) is defined as hypertensive blood pressure values which first occur after the 20th week of gestation with new-onset proteinuria. In the absence of proteinuria, other diagnostic features that are typical for organ dysfunction associated with preeclampsia, which include impaired liver function, thrombocytopenia, severe persistent pain in the right upper abdomen, renal insufficiency, pulmonary edema, fetal growth restriction (FGR), headache, and new-onset of visual symptoms, may confirm the diagnosis [10,17,18].

The pathogenesis of PE is not completely clarified yet. The most widespread hypothesis is based on an abnormal placentation [19]. The uterine arteries play a key role in the vascularization of the uterus. They branch into the arcuate arteries within the superficial myometrium, which give rise to radial arteries. These, in turn, lead to the basal arteries and terminate as spiral arteries. Throughout pregnancy, these spiral arteries provide blood flow to the intervillous space of the placenta. Remodeling of the uteroplacental vascular system occurs throughout different stages of pregnancy. This remodeling is essential for the reduction in vascular resistance to occur, which enables an increase in uterine blood flow, and is therefore necessary for successful placental implantation and normal placental function [20,21]. In some pregnancies, impaired remodeling of the maternal spiral arteries occurs early in gestation [19]. This causes a dysfunctional uteroplacental circulation, hypoxia, and placental apoptosis and necrosis [22,23,24]. Ultimately, oxidative stress causes endothelial dysfunction and an exuberant inflammatory response through the release of various angiogenic factors [22,25]. A maladaptive response with a reduced dilatory ability causes hypertension and increased capillary leakage, which induces proteinuria [22,26].

It is assumed that the decreased vascular capacitance and increased uteroplacental vascular resistance cause changes in the waveform of the uterine arteries which can be detected by Doppler examinations. Doppler studies of the uterine arteries allow a reproducible, non-invasive examination of placentation [21]. Several studies showed an association between abnormal Doppler examinations in the first and second trimester and the development of PE or FGR in ongoing pregnancy [27,28,29,30,31,32,33]. Initial studies in twin pregnancies have demonstrated a similar association; however, the mean uterine artery pulsatility index was significantly lower in these studies [14,34,35,36]. In their meta-analysis, Cao et al. reported a sensitivity of 65% and a specificity of 88% for the prediction of preeclampsia [37]. In contrast, studies in twin pregnancies reported lower sensitivities (33.3–36.4%) and higher specificities (88–96.7%) [38,39].

Early detection of impaired placental perfusion and development of PE at an early stage of pregnancy may be of great interest due to its potential to enable a prophylactic treatment at a time when the effect has been demonstrated.

The aim of this work was to investigate uterine artery Doppler examinations in twin pregnancies at the first and second trimester in relationship to hypertensive disorders.

## 2. Materials and Methods

This was a retrospective study to evaluate the use of the mean uterine artery pulsatility index (UTPI) in gestational weeks 11^+0^–13^+6^ and 19^+0^–22^+6^ for risk evaluation regarding the onset of preeclampsia or other obstetric adverse outcomes in twin pregnancies, conducted at the Department of Obstetrics and Gynecology of the Medical University of Vienna, a tertiary care center, over a study period of 4 years. Out of the 585 women with uncomplicated twin pregnancies above the age of 18 years who received measurements of their UTPI in gestational weeks 11^+0^–13^+6^, a total of 31 patients were excluded. Thirteen of them did not have a documented ultrasound scan at gestational weeks 19^+0^–22^+6^, five women had missing UTPI measurements at gestational weeks 11^+0^–13^+6^ or 19^+0^–22^+6^, three women had missing fetal outcome data, five women had missing maternal characteristics data, one woman had an abortion with mifepristone at gestational week 22^+6^, one woman had feticide of one fetus due to trisomy 21, three women had a miscarriage. The remaining 554 patients had measurement of both uterine arteries at gestational weeks 11^+0^–13^+6^ and 19^+0^–22^+6^, and complete maternal characteristics and fetal outcome data. Ultrasound was performed with Voluson E8 Expert, a transabdominal RAB 4-8-D convex probe was used–alternatively, and ultrasound was conducted with General Electric Healthcare E10 with a transabdominal RM 6C convex probe. In addition to the first-trimester screening, ultrasound examinations according to the clinic’s operating standard for twin pregnancies were conducted. Monochorionic twins were examined every two weeks from 16^+0^ weeks of gestation onwards and dichorionic twins were examined every four weeks. First-trimester screening was performed between 11^+0^ and 13^+6^ weeks of gestation, and standardized criteria according to the Fetal Medicine Foundation (London) were followed. At this appointment, chorionicity was determined by visualizing the lambda sign in DC twin pregnancies and the T-sign in MC twin gestations. Initially, gestational age was initially calculated regarding the first day of the last normal menstruation. By measuring the crown–rump-length (CRL) of the larger twin it was then adjusted. Additionally, the women received an anomaly scan between 20^+0^ and 23^+6^ weeks of gestation. Either transabdominal or transvaginal approach was conducted for measuring the pulsatility index (PI) of the uterine arteries. For transabdominal measurement, the uterine arteries were identified beside the cervix by high-velocity blood flow using color Doppler in the longitudinal plane. The angle of insonation was intended to be < 30° and the measurement was obtained as close as possible to the internal cervical os. The PI was measured when at least three identical waveforms were obtained. Transvaginal assessment of UTPI was done using the same principles. At 19^+0^–22^+6^ weeks, the PI of both uterine arteries was measured by transabdominal ultrasound. Data of included women with twin gestations were collected from Viewpoint^®^ software 5.6.28 (GE Healthcare, Wessling, Germany). The following data were extracted: age, weight, height, conception mode, gestational week at delivery, nicotine consumption, parity, placental abruption, stillbirth, UTPI in gestational weeks 11^+0^–13^+6^ and 19^+0^–22^+6^, chorionicity, preexisting chronic hypertension, gestational hypertension, early PE, late PE, HELLP, eclampsia, FGR, and intrauterine fetal death (IUFD).

Preeclampsia was defined in accordance with the American College of Obstetricians and Gynecologists as a newly onset blood pressure ≥140/90 mmHg measured over 20 weeks of gestation on two different occasions at least 4 h apart in women with a previously normal blood pressure, or a blood pressure ≥160/110 and proteinuria. Definition of proteinuria included ≥300 mg protein excretion in the 24 h urine collection, a protein/creatinine ratio of 0.3 mg/dL, or, alternatively, a urine dipstick reading of 2+ (this method was used only if other quantitative methods were not available). In women with newly diagnosed hypertension, preeclampsia was diagnosed if any of the following occurred—even in the absence of proteinuria: a platelet count below 100,000/μL, serum creatinine above 1.1 mg/dL, liver transaminase levels at least twice the normal limit, pulmonary edema, FGR, or neurological symptoms such as visual disturbances or headaches. Gestational hypertension was defined as blood pressure ≥140/90 mmHg in the absence of significant proteinuria after 20 weeks of gestation [10]. The primary endpoint of this study was the development of PE, which was subdivided into early and late PE. Early PE is defined as PE diagnosis before 34^+0^ weeks of gestation and late PE as PE diagnosis after 34^+0^ weeks of gestation. The secondary endpoint was defined as occurrence of adverse pregnancy outcome, which includes preterm birth <32^+0^ weeks of gestation, PE, gestational hypertension, FGR, stillbirth, placental abruption, HELLP syndrome, and eclampsia. None of the patients received low-dose aspirin for preeclampsia prophylaxis. When indicated, patients with hypertensive pregnancy disorders received antihypertensive treatment (e.g., methyldopa, enalapril, nifedipine) for blood pressure control, in accordance with a local clinical guideline. In cases of preterm birth before 34^+0^ weeks of gestation, patients received antenatal corticosteroids for fetal lung maturation, and, in cases before 32^+0^ weeks, they received neuroprotection with magnesium sulfate. The Ethical Review Board of the Medical University of Vienna (EK Nr: 2071/2018) approved the conduction of this study. It was performed according to the standards of the Helsinki Declaration. Declaration of consent was obtained at registration according to the department’s operating standards. Data analysis was conducted using SPSS 26.0 for Mac (SPSS Inc., Chicago, IL, USA). A Kolmogorov–Smirnov test was used to verify data distribution. Variables that were normally distributed are presented as means (± standard deviation (SD)), categorical data as percentages. Further statistical tests including Chi-square test, McNemar test, and logistic multiple regression analyses were used. Logistic regression was obtained using the backward selection with likelihood ratio. The Hosmer and Lemeshow test was used. In addition, a receiver operating characteristics (ROC) curve was created to calculate sensitivity and specificity. The cut-off for mean PI was separately defined according to the Youden index. We calculated sensitivity, specificity, and relative risk with 95% confidence interval (95% CI). All tests were two tailed, *p*-value ≤ 0.05 was considered statistically significant.

## 3. Results

Out of the 554 women included in this retrospective analysis, a total of 51 women (9.2%) developed PE. Twelve women (2.2%) developed early PE and 39 patients (7.0%) developed late PE. An adverse pregnancy outcome (including preterm birth < 32^+0^ weeks of gestation, PE, gestational hypertension, FGR, stillbirth, placental abruption, HELLP syndrome, and eclampsia) occurred in 147 women (26.5%). Clinical characteristics of the subgroups (PE, early PE, late PE, and adverse pregnancy outcome) are presented in Table 1 and Table 2.

In the observed monochorionic twin pregnancies, 14.7% developed PE, compared to 9.8% in the dichorionic twin gestations. The mean maternal age was 32 years in the control group and 34 in the PE cohort. The median body mass index (BMI) was 22.7 kg/m^2^ in the women without PE and 23.5 kg/m^2^ in those with PE. The control group consisted of 57.1% nullipara women, whereas the PE group included 74.5%. The control cohort had 63.4% spontaneous conceptions. Assisted reproductive technology (ART) was used in 36.6%. In the PE group, 47.1% of the women had a spontaneous conception and ART was performed in 52.9% of the patients. The group of women without PE had 70.0% DC gestations and 30.0% MC twins. The PE group consisted of 78.4% DC gestations and 21.6% MC twins. The median gestational age of the neonates that were delivered was 36.7 weeks in the control group, whereas it was 36.0 weeks in the PE cohort.

**Figure 1:**
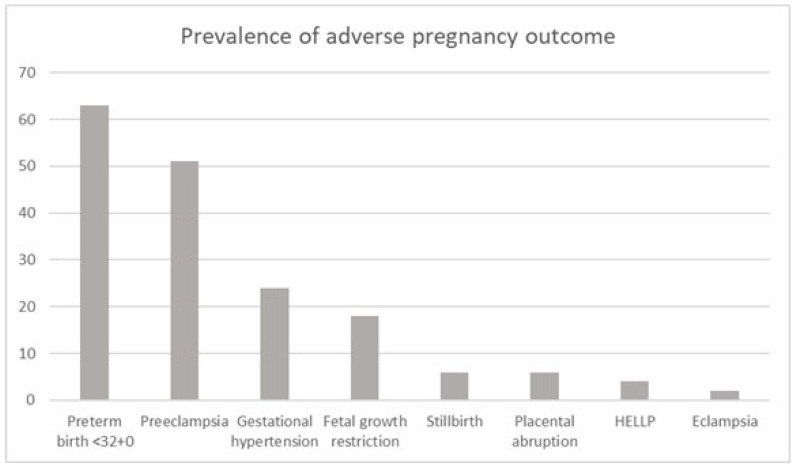
Cumulative prevalence (percentage) in the adverse pregnancy outcome group.

Patient characteristics of the subgroup affected by adverse pregnancy outcome (including preterm birth <32^+0^ weeks of gestation, PE, gestational hypertension, FGR, stillbirth, placental abruption, HELLP syndrome, and eclampsia) are outlined in Table 2. The prevalence of the individual parameters within the adverse pregnancy outcome group is shown in Figure 1.

As seen in Table 3, the median mean UTPI in gestational weeks 11^+0^–13^+6^ in the women without PE was 1.23 (range of 0.36–2.97) and it was 1.26 (range of 0.54–2.57) in the women with PE. In gestational weeks 19^+0^–22^+6^, the women without PE had a median mean UTPI of 0.8 (range of 0.33–1.78) and the women with PE had a mean UTPI of 0.81 (range of 0.47–1.56).

The median mean UTPI in gestational weeks 11^+0^–13^+6^ in the MC twins was 1.38 (range 0.54–2.45) and it was 1.24 (range 0.36–2.97) in the DC twins. In gestational weeks 19^+0^–22^+6^, the MC pregnancies had a median mean UTPI of 0.88 (range 0.47–1.76) and the DC pregnancies had a median mean UTPI of 0.82 (range 0.33–1.78) (Table 4).

**Figure 2:**
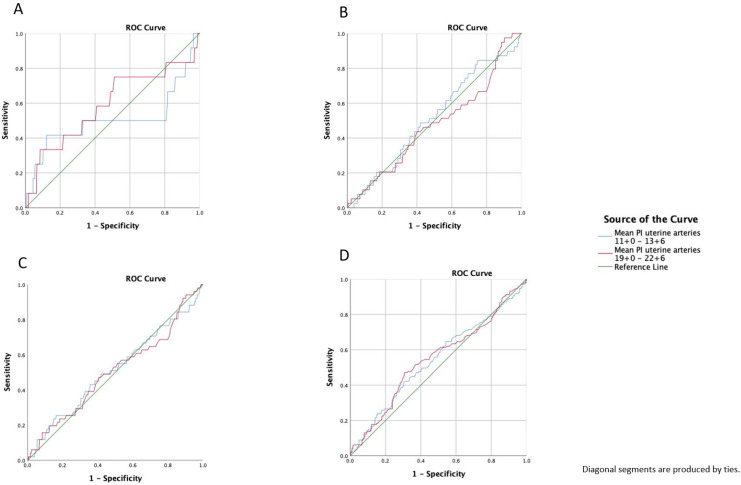
Receiver operating characteristics curve (ROC) prediction (**A**): early preeclampsia, (**B**): late preeclampsia, (**C**): preeclampsia, and (**D**): adverse pregnancy outcome with a mean pulsatility index of the uterine arteries at 11^+0^–13^+6^ and 19^+0^–22^+6^ weeks of gestation; adverse pregnancy includes preterm birth <32^+0^ weeks of gestation, PE, gestational hypertension, FGR, stillbirth, placental abruption, HELLP syndrome, and eclampsia.

The detection rates of pregnancies complicated by PE or an adverse pregnancy outcome (including preterm birth <32^+0^ weeks of gestation, PE, gestational hypertension, FGR, stillbirth, placental abruption, HELLP syndrome, and eclampsia) were calculated, and ROC curves that predicted PE, early PE, late PE, and adverse obstetric outcomes in gestational weeks 11^+0^–13^+6^ and 19^+0^–22^+6^ were constructed. None of the individual adverse pregnancy outcomes, except for PE, were significantly associated with the uterine artery Doppler measurements. We calculated the area under the curve (AUC) for each curve. For the prediction of PE, the AUC was 0.512 for gestational weeks 11^+0^–13^+6^ and 0.504 for gestational weeks 19^+0^–22^+6^. The AUC for prediction of early PE by UTPI was 0.502 for the gestational weeks 11^+0^–13^+6^ and 0.589 for the gestational weeks 19^+0^–22^+6^. For late PE, the AUC was 0.515 for 11^+0^–13^+6^ weeks of gestation and 0.467 for 19^+0^–22^+6^ weeks of gestation. For adverse pregnancy outcomes, the AUC at 11^+0^–13^+6^ weeks of gestation was 0.543 and it was 0.547 for the weeks 19^+0^–22^+6^ (Figure 2).

The Youden index was defined for all points on the ROC curves for the prediction of PE, alongside the mean UTPI for gestational weeks 11^+0^–13^+6^ and 19^+0^–22^+6^. The optimum cut-off for the mean UTPI was selected for the gestational weeks 11^+0^–13^+6^ and 19^+0^–22^+6^, using the highest Youden index. For the gestational weeks 11^+0^–13^+6^, the cut-off was set at a mean UTPI of 1.682 and, for the gestational weeks 19^+0^–22^+6^, it was set at 1.187.

The sensitivity, specificity, positive predictive value, negative predictive value, and likelihood ratio with a 95% confidence interval for PE, early PE, late PE, and adverse pregnancy outcome (including preterm birth <32^+0^ weeks of gestation, PE, gestational hypertension, FGR, stillbirth, placental abruption, HELLP syndrome, and eclampsia) were calculated for gestational weeks 11^+0^–13^+6^ and 19^+0^–22^+6^, as shown in Table 5 and Table 6.

In the gestational weeks 11^+0^–13^+6^, there was a 1.5-fold increased risk for developing PE with a mean UTPI above the set cut-off. The risk of early PE was increased 2.5-fold, and that of an adverse pregnancy outcome was increased 1.5-fold. For late PE, there was only a slightly increased risk, by 1.2-fold, if the mean UTPI was above the cut-off.

In the gestational weeks 19^+0^–22^+6^, the risk of developing PE was increased 2-fold if the mean UTPI was above the set cut-off. For early PE, the risk was increased 4-fold and, for adverse pregnancy outcome, it was increased 1.5-fold. Again, there was only a slightly increased risk (1.1-fold) of developing late PE with a mean UTPI above the cut-off.

To compare testing in the gestational weeks 11^+0^–13^+6^ and 19^+0^–22^+6^, crosstables and the McNemar test was used (Table 7).

The McNemar test is highly significant (*p* < 0.001) for measurement of the mean cut-off UTPI in gestational weeks 11^+0^–13^+6^ and 19^+0^–22^+6^. Therefore, there is a difference between these measurements.

For women who developed PE, the McNemar test for measurement of the mean cut-off UTPI in gestational weeks 11^+0^–13^+6^ and 19^+0^–22^+6^ was not statistically significant (*p* = 0.18). There seems to be no link between the first and second measurements in women with PE.

The McNemar test is highly significant (*p* < 0.001) for women without PE regarding the mean cut-off UTPI in gestational weeks 11^+0^–13^+6^ and 19^+0^–22^+6.^ Thus, there is a link between measurements of the UTPI in these gestational weeks.

The following parameters were included for the regression analysis: maternal age, maternal BMI, smoking habits, nulliparity, and UTPI in gestational weeks 11^+0^–13^+6^ > 1.682 and 19^+0^–22^+6^ > 1.187. The analysis revealed that increases in the maternal age, maternal BMI, smoking habits, and/or mean PI of the uterine arteries at both times (gestational weeks 11^+0^–13^+6^ and 19^+0^–22^+6^) were associated with preeclampsia, early preeclampsia, late preeclampsia, and/or adverse pregnancy outcome (Table 8).

## 4. Discussion

In our study, we investigated the predictive value of the mean UTPI within the first and second-trimester screening for preeclampsia and adverse pregnancy outcomes in twin pregnancies. Early preeclampsia can be identified more accurately than late preeclampsia. A combination of the mean UTPIs of the first and second trimesters could most effectively forecast preeclampsia.

Various studies have investigated the use of uterine artery Doppler examinations to predict preeclampsia, growth restriction, and unfavorable pregnancy outcomes in singleton pregnancies [27,28,31,40,41,42,43,44,45]. Similar studies have been conducted on twin pregnancies [14,35,36,38,39,46,47,48,49,50]. The current study demonstrates that the mean UTPI at gestational weeks 11^+0^–13^+6^ and 19^+0^–22^+6^ is lower than previously reported in singleton pregnancies [40,51]. This has already been described by several other authors [14,35,36,38,39,46,48,49]. Rizzo et al. assume that the lower PI values result from a bigger placenta implantation site and altered maternal hemodynamics in twin pregnancies [14]. According to Geipel et al., the majority of UTPI values in twin pregnancies are below the 50th percentile of the reference range for singleton pregnancies [46]. Therefore, using reference values from singleton pregnancies could lead to a false-negative classification of patients at risk. Queirós et al. identified all cases of early-onset preeclampsia using the UTPI 90th percentile for twins. However, the detection rate dropped to 50% when singleton reference values were used [36]. Therefore, the use of twin-specific UTPI reference values for risk stratification should be considered.

In contrast to previous research, this study defined the cut-off for an elevated mean UTPI using the Youden index rather than the 95th percentile. The cut-off for the gestational ages 11^+0^–13^+6^ and 19^+0^–22^+6^ was set at 1.683 and 1.188, respectively. So far, no reference ranges for an elevated mean UTPI in twin gestations at gestational weeks 11^+0^–13^+6^ have been published. The first-trimester cut-off value described is lower than those used by Pilalis et al. and Gómez et al., who reported the 95th percentile of UTPI at 2.24–2.52 in singleton pregnancies [40,52]. Remarkably, our cut-off value used for gestational weeks 19^+0^–22^+6^ is comparable to the reference charts for the mean UTPI in twin pregnancies published by Geipel et al. They described an elevated mean UTPI above the 95th percentile, with values of 1.259 at 20 weeks, 1.206 at 21 weeks, 1.161 at 22 weeks, and 1.124 at 23 weeks. This results in an average cut-off for the mean UTPI in gestational weeks 19^+0^–22^+6^ of 1.188, which is nearly identical to the one used in our study [46].

Regardless of whether preeclampsia screening is performed between weeks 11^+0^ and 13^+6^, between weeks 19^+0^ and 22^+6^, or through a combination of both, our study shows that early preeclampsia can be predicted with the highest accuracy. Previously performed studies already outlined that the uterine artery Doppler is especially effective in identifying those women who will develop early-onset preeclampsia in comparison to late-onset preeclampsia [14,27,28,41,47,48].

We observed sensitivities and specificities of 41.7% and 83.4% at 11^+0^–13^+6^ weeks, 33.3% and 91.5% at 19^+0^–22^+6^ weeks, and 33.3% and 95.6% when using a combination of both. Recent studies on twin pregnancies utilizing twin references reported sensitivities of 33.3–36.4% and specificities of 88–96.7% for the prediction of preeclampsia [38,39]. The calculated likelihood ratio for a positive test for early preeclampsia was lower in gestational weeks 11^+0^–13^+6^ compared to weeks 19^+0^–22^+6^ and when both were combined. As the accuracy of predicting preeclampsia improves throughout pregnancy, using UTPI values from earlier gestational ages could result in lower predictive values. The combination of the two examinations resulted in the best likelihood ratio. This study is the first to present these results. Furthermore, in our study, we did not find any differences in the mean UTPI values concerning the chorionicity of the twins. Most previous studies have reported similar results [14,39,46,47,48], with only two suggesting the opposite [34,50].

There is broad consensus that multiple pregnancies carry a higher risk of hypertensive pregnancy disorders [11,13,16,53]. Identifying twin pregnancies at the highest risk of preeclampsia would facilitate more targeted and intensive prenatal care. We agree with Rizzo et al. that an isolated use of UTPI may have a limited role in the prediction of preeclampsia in twin pregnancies [14]. Recent studies on multi-marker screening models in twin pregnancies demonstrate good screening performance but show lower sensitivities and higher screen-positive rates compared to singleton pregnancies [34,54,55,56]. The question is whether screening after 16^+0^ weeks of gestation is too late for the stratification of low-dose aspirin therapy. Only one meta-analysis on preeclampsia prevention revealed a certain effect of aspirin in twin pregnancies. The results were inconsistent with those in singleton pregnancies, demonstrating a reduction in mild but not severe preeclampsia. Furthermore, the reduction was achieved when aspirin was initiated after 16^+0^ weeks of gestation but not before [57]. For this reason, a two-tailed test with better predictive value could more effectively identify patients who may benefit from aspirin therapy and intensive prenatal care.

As far as we know, we are the first to use a combination of the mean UTPI values in the first and second trimesters to predict preeclampsia in twin pregnancies. We consider this, along with the relatively large number of participants, to be a strength of our study. Nevertheless, several limitations should be addressed. First, this was a single-center retrospective study, which limits the generalizability of our findings to other settings and populations. Another limitation of this study is that we could not include the mean arterial blood pressure, nor biochemical markers such as pregnancy-associated plasma protein A, soluble Fms-like tyrosine kinase-1, and placental growth factor, or their ratio, in combination with uterine artery Doppler measurements to create predictive models for the development of preeclampsia.

## 5. Conclusions

We conclude that the best prediction for early-onset preeclampsia in twin pregnancies is achieved by using a two-tailed screening approach that combines UTPI measurements taken between gestational weeks 11^+0^–13^+6^ and 19^+0^–22^+6^.

## Figures and Tables

**Table 1:** Patient characteristics of patients with preeclampsia, early preeclampsia, and late preeclampsia and control group.

Characteristic	Controls (N = 503)	Preeclampsia (N = 51)	Early Preeclampsia (N = 12)	Late Preeclampsia (N = 39)
age, y, *	31.8 (5.8)	34.0 (6.5)	34.6 (6.6)	33.8 (6.6)
**BMI, kg/m^2^, †**	22.7 (15.5–49.4)	23.5 (18.9–37.6)	29.1 (19.4–35.6)	23.5 (18.9–37.6)
**nulliparity, ‡**	287 (57.1)	38 (74.5)	8 (66.7)	30 (76.9)
**smoking, ‡**	49 (9.7)	6 (11.8)	3 (25.0)	3 (7.7)
**ART, ‡**	184 (36.6)	27 (52.9)	8 (66.7)	19 (48.7)
**chorionicity, ‡**				
**DC twins**	352 (70.0)	40 (78.4)	9 (75.0)	31 (79.5)
**MC twins**	151 (30.0)	11 (21.6)	3 (25.0)	8 (20.5)
**gestational weeks at delivery, †**	36.7 (23.0–41.1)	36.0 (28.7–38.9)	33.5 (28.7–35.7)	36.3 (30.4–38.9)

* Mean (standard deviation), † median (interquartile range), ‡ number (percent). y = year, BMI = body mass index; kg/m^2^ = kilogram per square meter; ART = assisted reproductive technology; DC = dichorionic; MC = monochorionic.

**Table 2:** Patient characteristics of patients with adverse pregnancy outcome and control group.

Characteristic	Controls (N = 407)	Adverse Pregnancy Outcome (N = 147)
age, y, *	31.8 (5.7)	32.5 (6.3)
**BMI, kg/m^2^, †**	22.7 (15.8–49.4)	23.3 (15.5–40.7)
**nulliparity, ‡**	221 (54.3)	104 (70.7)
**smoking, ‡**	37 (9.1)	18 (12.2)
**ART, ‡**	143 (35.1)	68 (46.3)
**chorionicity, ‡**		
**DC twins**	292 (71.7)	100 (68.0)
**MC twins**	115 (28.3)	47 (32.0)
**gestational weeks at delivery, †**	37.0 (30.3–39.9)	34.1 (23.0–41.1)

* Mean (standard deviation), † median (interquartile range), ‡ number (percent). y = year, BMI = body mass index; kg/m^2^ = kilogram per square meter; ART = assisted reproductive technology; DC = dichorionic; MC = monochorionic; adverse pregnancy includes preterm birth <32^+0^ weeks of gestation, PE, gestational hypertension, FGR, stillbirth, placental abruption, HELLP syndrome, and eclampsia.

**Table 3:** Pulsatility index for the uterine arteries for gestational weeks 11^+0^–13^+6^ and 19^+0^–22^+6^ in the control group and the preeclampsia group.

Characteristics	Controls (N = 503)	Preeclampsia (N = 51)	*p*
UTPI at gestational weeks 11^+0^–13^+6^, *	1.23 (0.36–2.97)	1.26 (0.54–2.57)	0.77
UTPI at gestational weeks 19^+0^–22^+6^, *	0.80 (0.33–1.78)	0.81 (0.47–1.56)	0.93

* Median (range); UTPI = pulsatility index of the uterine arteries.

**Table 4:** Pulsatility index of the uterine arteries for gestational weeks 11^+0^–13^+6^ and 19^+0^–22^+6^ in monochorionic and dichorionic twin pregnancies.

Characteristics	MC (N = 162)	DC (N = 392)	*p*
UTPI at gestational weeks 11^+0^–13^+6^, *	1.38 (0.54–2.45)	1.24 (0.36–2.97)	<0.01
UTPI at gestational weeks 19^+0^–22^+6^, *	0.88 (0.47–1.76)	0.82 (0.33–1.78)	<0.01

* Median (range); MC = monochorionic, DC = dichorionic, UTPI = pulsatility index of the uterine arteries.

**Table 5:** The predictive value of a mean uterine artery pulsatility index >1.682 at gestational weeks 11^+0^–13^+6^ for preeclampsia, early preeclampsia, late preeclampsia, and adverse pregnancy outcome.

Characteristics	Sensitivity	Specificity	PPV	NPV	LR+	95% CI
**preeclampsia** **(n = 51)**	25.5%	83.7%	13.7%	91.7%	1.6	0.94–2.60
**early preeclampsia** **(n =12)**	41.7%	83.4%	5.3%	98.5%	2.5	1.25–5.03
**late preeclampsia** **(n =39)**	20.5%	83.1%	8.4%	93.2%	1.2	0.64–2.32
**adverse pregnancy outcome** **(n = 147)**	23.1%	85.0%	35.8%	75.4%	1.5	1.06–2.24

PPV = positive predictive value; NPV = negative predictive value; LR+ = likelihood ratio of positive test result; CI = confidence interval; adverse pregnancy outcome includes preterm birth <32^+0^ weeks of gestation, PE, gestational hypertension, FGR, stillbirth, placental abruption, HELLP syndrome, and eclampsia.

**Table 6:** The predictive value of a mean uterine artery pulsatility index >1.187 at gestational weeks 19^+0^–22^+6^ for preeclampsia, early preeclampsia, late preeclampsia, and adverse pregnancy outcome.

Characteristics	Sensitivity	Specificity	PPV	NPV	LR+	95% CI
**preeclampsia** **(n = 51)**	15.7%	91.7%	16.0%	91.5%	1.9	0.93–3.78
**early preeclampsia** **(n = 12)**	33.3%	91.5%	8.0%	98.4%	3.9	1.68–9.16
**late preeclampsia** **(n = 39)**	10.3%	91.0%	8.0%	93.1%	1.1	0.44–3.02
**adverse pregnancy outcome** **(n = 147)**	12.2%	92.1%	36.0%	74.4%	1.6	0.90–2.69

PPV = positive predictive value; NPV = negative predictive value; LR+ = likelihood ratio of positive test result; CI = confidence interval; adverse pregnancy outcome includes preterm birth <32^+0^ weeks of gestation, PE, gestational hypertension, FGR, stillbirth, placental abruption, HELLP syndrome, and eclampsia.

**Table 7:** Crosstable for McNemar test for mean pulsatility index of the uterine arteries (UTPI) above the cut-off (1.188) in gestational weeks 19^+0^–22^+6^ and mean UTPI above the cut-off (1.683) in gestational weeks 11^+0^–13^+6^.

			Mean UTPI > 1.683 at Gestational Weeks 11^+0^–13^+6^		
			Yes	No	Total
**mean UTPI > 1.188** **at Gestational weeks 19^+0^–22^+6^**	**Yes**	**Count**	27	23	50
		**% within mean UTPI > 1.188 at gestational weeks 19^+0^–22^+6^**	75%	46%	100%
		**% within mean UTPI > 1.683 at gestational weeks 11^+0^–13^+6^**	28.4%	5%	9%
	**No**	**Count**	68	436	504
		**% within mean UTPI > 1.188 at gestational weeks 19^+0^–22^+6^**	75%	86.5%	100%
		**% within mean UTPI > 1.683 at gestational weeks 11^+0^–13^+6^**	71.6%	95%	91%
**Total**		**Count**	95	459	554
		**% within mean UTPI > 1.188 at gestational weeks 19^+0^–22^+6^**	75%	82.9%	100%
		**% within mean UTPI > 1.683 at gestational weeks 11^+0^–13^+6^**	100%	100%	100%

UTPI = pulsatility index of the uterine arteries.

**Table 8:** Predictors of adverse obstetric outcomes.

Variable	OR	95% CI	*p* Value
**prediction of preeclampsia**			
**maternal age**	1.07	1.02–1.12	0.01
**maternal BMI**	1.07	1.01–1.13	0.015
**nulliparity**	2.75	1.38–5.47	0.004
**mean UTPI at gestational weeks 11^+0^–13^+6^ > 1.682 and 19^+0^–22^+6^ > 1.187**	2.84	1.05–7.77	0.03
**prediction of early preeclampsia**			
**maternal BMI**	1.1	1.00–1.20	0.04
**mean UTPI at gestational weeks 11^+0^–13^+6^ > 1.682 and 19^+0^–22^+6^ > 1.187**	10.13	2.8–36.73	<0.001
**prediction of late preeclampsia**			
**maternal age**	1.06	1.00–1.12	0.04
**nulliparity**	2.53	1.17–5.44	0.02
**prediction of adverse pregnancy outcome**			
**nulliparity**	2.28	1.53–3.58	<0.001
**mean UTPI at gestational weeks 11^+0^–13^+6^ > 1.682 and 19^+0^–22^+6^ > 1.187**	2.91	1.31–6.64	0.009

OR = odds ratio, BMI = body mass index, CI = confidence interval, UTPI = pulsatility index of the uterine arteries; adverse pregnancy outcome includes preterm delivery before 32^+0^ weeks of gestation, preeclampsia, gestational hypertension, fetal growth restriction, stillbirth, placental abruption, HELLP syndrome, and eclampsia.

## Data Availability

The data are not publicly available due to local data protection guide-lines.

## Abbreviations

The following abbreviations are used in this manuscript:

UTPI	uterine artery pulsatility index
PE	preeclampsia
MC	monochorionic
DC	dichorionic
PI	pulsatility index
FGR	fetal growth restriction
SD	standard deviation
CRL	crown-rump-length
ROC	receiver operating characteristics
CI	confidence interval
AUC	area under the curve
ART	assisted reproductive technology
IUFD	intrauterine fetal death
BMI	body mass index
NPV	negative predictive value
PPV	positive predictive value
LR+	likelihood ratio of positive test result
